# Piloting an approach to scab control on Welsh sheep farms

**DOI:** 10.1002/vro2.30

**Published:** 2022-03-12

**Authors:** Neil Paton, Stewart Burgess, Kathryn Bartley, John Griffiths, Edgar Jones

**Affiliations:** ^1^ Veterinary Epidemiology Economics and Public Health The Royal Veterinary College Hawkshead Lane North Mymms Hatfield Hertfordshire UK; ^2^ Moredun Research Institute Pentlands Science Park Bush Loan Edinburgh Midlothian Scotland UK; ^3^ Coleg Sir Gar Graig Campus Sandy Road Pwll Wales UK

## Abstract

**Introduction:**

Sheep scab caused by *Psoroptes ovis*, is a disease of concern to many stakeholders in Wales due to its welfare implications. There are good diagnostic tests and treatments available to deal with the disease. Even so, it remains a problem in Welsh flocks. As such a coordinated approach is required to deal with this issue in a more sustainable manner.

**Pilot design:**

Sheep scab positive ‘index’ farms were initially diagnosed using a skin scrape to identify *P. ovis* mites. Contiguous farms were identified and antibody responses used to confirm onward infestation. All infested farms were treated by either dipping with an organophosphate (OP) dip or injecting with a licensed macrocyclic lactone (ML) product depending on farmer choice.

**Results:**

Three positive ‘index’ farms were identified along with 12 contiguous properties. Positive serological responses were observed in seven of the 12 contiguous farms; four of which were treated by OP dip and three by an injectable ML product.

**Discussion:**

To avoid reinfestation of treated farms, dealing with disease on contiguous properties is crucial. Through the project coordinating team, three local outbreaks of scab were dealt with in a short space of time with appropriate diagnosis and treatment being carried out. Some farmers were uncooperative and strategies such as providing additional external support and veterinary involvement might alleviate these issues in the future. This coordinated approach is recommended to veterinary surgeons in the field when dealing with scab on farm.

## INTRODUCTION

Sheep scab is caused by infestation of the flock with the ectoparasitic mite *Psoroptes ovis*. Infestation causes pruritus due to hypersensitivity to components of the mite faeces deposited on the skin. This can be severe enough to cause significant self‐trauma, limiting ‘normal’ behaviours such as feeding and lead to productivity losses, significant welfare concerns and death in extreme cases.^1^ The disease also causes significant productivity losses through effects including weight loss^2^ with the annual cost of the disease to the UK sheep industry estimated between £78 and 202 million.^3^

The prevalence of disease in the Welsh national flock is difficult to determine due to a reluctance to report, which has traditionally been ascribed to the stigma associated with the disease. A number of studies have attempted to determine how many farms are likely to be infested and estimates range from 8.6% to 36% of farms, though there is considerable uncertainty due to under reporting of the disease.^4^

The welfare implications and the large number of farms affected mean that controlling sheep scab is a priority of the Welsh Animal Health and Welfare Framework Group (AHWFG).^5^ A task and finish group started work to develop an eradication plan and this proposal was submitted to the Welsh Government for consideration. In January 2019 it was announced that £5 million would be committed to sheep scab eradication in Wales. There are significant challenges to dealing with this disease in Wales as acknowledged by the Welsh AHWFG. Wales has a relatively large sheep population with a large proportion of common grazing and seasonal movement of sheep to and from high and low ground, all making disease control and eradication difficult.

It is suspected that a contributing factor in the high prevalence is the misdiagnosis of the disease leading to inappropriate treatment. Many farmers rely on clinical signs when making treatment decisions for sheep scab, however clinical inspection of sheep is often insufficient to reliably differentiate between infestation with *P. ovis* and other agents of skin disease, meaning that sheep are often inappropriately treated. The presence of the mite can be detected by scraping lesions and examining the scraped material under a microscope. This can be useful in identifying an active infestation if live mites are found, however, the sensitivity of the method is highly variable.^6^ As an alternative or to support the diagnosis the detection of host antibodies with specificity to the mite antigen, Pso o 2 can be used to identify exposure, however, interpreting the results accurately requires an understanding of the clinical picture on the farm where the samples were taken.^7^, ^8^

Treatment of scab relies heavily on two classes of products, the macrocyclic lactones (ML) injectable products and organophosphate (OP) through plunge dipping.^9^ There are concerns such as poor dipping technique and misapplication via unverified delivery methods, such as jetters and shower units, leading to reduced efficacy. Dipping also has significant environmental risks associated with its use^10^ and concerns around operator safety.^11^ The alternative is the use of the ML injectable products, which are safer for the operator although they still have environmental risks^12^ and may hasten the development of resistance to ML‐based anthelmintics, which are heavily relied upon for the control of gastro‐intestinal nematodes in sheep. Of concern are reports of resistance to ML injectable products in *P. ovis* mites in the UK^13^, ^14^ which raises the concern that there may be only one viable treatment option in the future.

Working with Welsh Government and the Welsh AHWFG, it was recognised that a new approach to control the disease was required, which should be field tested in a pilot study. In an effort to keep the industry engaged and invested a proof‐of‐concept project was designed and implemented, aiming to increase awareness among stakeholders, trialling this innovative approach in the field.

## MATERIALS AND METHODS

### Pilot design

Veterinary practices from three regions—south, mid and north Wales were contacted in order to recruit farms from different parts of Wales. In each region an ‘index’ farm infested with *P. ovis* was identified by a local veterinary practice. Farms were limited to about 200 animals, with farms of similar size, to support treatment of all animals on the properties recruited to the programme.

The index farm was asked for permission to contact neighbouring properties and all identifying data were retained by the technical officer. No other member of the project team was able to identify specific farms.

To confirm the presence of *P. ovis* a diagnostic skin scrape sample was sent to a commercial laboratory (Animal and Plant Health Agency, APHA) for confirmation and definitive diagnosis of sheep scab. If a report was held by the practice at the time of enquiry this was also considered suitable. Once confirmation was received the veterinary surgeon and farmer worked to identify contiguous farms to the affected unit. Each contiguous farm was risk assessed and if contact between sheep on each farm was plausible, the owner was approached with an offer of diagnosis and subsequent treatment if required. Farms were considered at risk if the fields next to the ‘index’ farm had been used to graze sheep, the type of fencing (double or single) was not considered sufficient to stop spread for the purposes of this project. Farms separated by a river, road unused for sheep movement in the previous 6 months, or forestry, were considered not at risk. In all cases it was considered better to test if in doubt about the level of exposure.

### Sheep scab ELISA testing

On contiguous farms the diagnosis of sheep scab was made through the *P. ovis* antibody ELISA test, with 12 sheep being tested from each management group or flock. The samples for antibody testing were sent to the Moredun Research Institute and tested according to published protocol.^7^, ^8^, ^15^ The veterinary surgeons were able to test multiple management groups on a single farm (×12 test animals from each) if these were managed separately, for example a housed group and a hill group on a single farm. If a single animal was antibody positive in any group, then the farm was considered to be infested. If clinical inspection suggested scab then a skin scrape was carried out on the suspected sheep, as an alternative to blood sampling.

### Treatment of positive flocks

Due to the pilot project being carried out at lambing time and the presence of heavily pregnant sheep which should ideally not be dipped, the treatment offered initially was a single injection with moxidectin 2% (Cydectin LA 2%; Zoetis, Leatherhead, Surry, UK) for all suitable animals and doramectin (Dectomax; Elanco, Hook, Hampshire, UK) for lambs under 15 kg, as indicated by the product data sheets. At farm level, treatment was carried out within 2 weeks of diagnosis.

## RESULTS

Three ‘index’ farms (A, B and C) with suspected cases of sheep scab were identified through local veterinary practices and skin scrape samples confirmed infestation with live *P. ovis* mites. The three farms had direct links to 12 contiguous properties (five, five and two, respectively—see Table 1). It was possible for all three ‘index’ farms to identify the owners of 100% of the adjoining land and to find the contact details of all farms involved.

**TABLE 1:** Contiguous farm numbers, sheep scab ELISA data and treatment outcomes for each index farm

Index farm	Number of contiguous farms	Number of contiguous farms tested	Number of sheep on all farms in cluster^a^	Number of sheep sampled on contiguous farms	Number of farms positive by sheep scab ELISA	Number of farms where sheep injected with macrocyclic lactone product^a^	Number of farms where sheep dipped with organophosphate product^a^	Number of sheep treated on all farms in the cluster^a^
A	5	5	2349	60	2	3	0	420
B	5	4	489	38	2		3	425
C	2	0	406	0	None tested	1	0	412
Total	12	9	3244	98	4	4	3	1257 (38%)

^a^ Includes ‘index’ farm.

Of the 12 contiguous properties, 10 were willing to engage in the pilot project. A further farmer, while willing to participate, was unable to gather the ewes identified for sampling. Another farm did sample the ewes but technical issues prevented analysis being carried out on this farm and management practices did not allow a second sampling session because the flock was away on rented ground.

Of the nine contiguous farms that were tested with the sheep scab ELISA (Clusters A and B, Table 1), four were identified as being positive (at least one animal testing positive in the flock/group screen), indicating exposure to *P. ovis*. This led to a total of seven farms requiring treatment for sheep scab, consisting of the three ‘index’ cases and the four positive contiguous properties. No infestations on contiguous farms were identified through clinical inspection and examination of skin scrapings.

The project offered financial support to treat 100% of the infested flocks. Four of the farms accepted the option of treating with a ML injectable product; while three others requested the option of OP dipping their animals. This led to the treatment of 1256 (38.7%) sheep out of a total of 3244 across the 15 properties (three ‘index’ farms and 12 contiguous properties).

This led to the treatment of 1256 (38.7%) sheep, that is, all of the sheep on the farms found to be infested, out of a total of 3244 across the 15 properties (three ‘index’ farms and 12 contiguous properties).

The first farm was diagnosed in early February 2021 and all work, including treatment was completed by the end of March 2021.

## DISCUSSION

The approaches discussed in this manuscript were based on plans previously submitted to the Welsh Government by some of the authors.^16^ The structure was to identify through self‐reporting an index farm with sheep scab. This would then lead to the identification of a group of contiguous farms and the owners and managers of these properties would be contacted and group meetings arranged. In these group meetings the gathering, sampling and ultimately treatment plans would be discussed and arranged. This approach has not been used in Wales before and the response by farmers to the concept was tested with three groups of farms.

Three properties were identified as being infested with *P. ovis* through veterinary practices. Permission from the farmer of the three index farms to contact neighbouring farmers was sought by the technical officer and no identifiable data were made available to other members of the team.

These properties were surrounded by a further 12 properties. Of the 12 surrounding properties, nine were able to cooperate fully with the programme and three did not. Nine properties had sheep that underwent antibody testing . Four were found to be positive through this testing. None of the surrounding properties had sheep with clinical signs. This lead to a total of seven farms that were positive (three initially scaped and four antibody positive). Less than half of the sheep on the farms in these clusters required treatment.

It was reasonably straight forward to identify three farms for the pilot where the farmers were willing to cooperate. This, along with APHA offering free testing of skin scrape samples from farms with suspected sheep scab over the winter period, suggests that the recruitment of farms and engagement in a wider roll out of a sheep scab control project would be possible. This project was carried out in the middle of the lambing period, which is a busy time for sheep keepers with an increased population of sheep, including smaller and more vulnerable animals. Even so, this did not seem to be a barrier to engagement with the farms involved in the project.

When deciding a farm was at risk from infestation from neighbouring properties, the decision was made that roads, rivers and forestry were likely to be sufficient barriers, whereas double fencing was not. Further work to determine whether this decision was valid is required to determine whether the appropriate amount of contiguous property testing was done. The contiguous farms were tested using the sheep scab ELISA. The presence of any antibodies from the ELISA test on farm was considered sufficient to diagnose an infestation considering that the index farm represented an increased risk for infestation on the contiguous properties. As stated four farms with evidence of infestation were found before clinical signs were noted.

Not all the farms that were classed as contiguous were able to go through all the steps in the programme. One farm while willing was not able to gather sheep within the timeframe of the pilot. A larger and wider project would hopefully overcome such constraints by appropriate planning to determine a farm‐specific solution. One farmer was initially willing and then withdrew consent due to personal issues, it may be possible to accommodate such farms in a larger project with solutions based on careful planning and local information.

The third farm was unwilling to cooperate with any initiative that was affiliated with the external agencies and particularly the Welsh Government. This is not unexpected; getting such farmers to cooperate is important for the success of control programmes and may be where the farmer's own veterinary surgeon, or other independent and trusted individuals, may be able to help to build trust and engagement.

Treatment of these farms was supported financially. It was anticipated by the project team that injectable product would be the most acceptable therapy for farmers due to environmental and operator safety concerns over the use of OP dips. The possibility of mismothering of lambs following dipping and avoiding dipping pregnant ewes were risks that farmers were considered likely to want to avoid. We intend to follow up the three farms that deployed OP dips to determine whether these risks actually occurred and at what level, in order to further inform advice should a wider roll out be undertaken. Although the offer was for injectable treatment a number of farmers requested dipping as a treatment option and this request was granted. Dipping was carried out by contract dippers engaged by the farmers directly. Farmers taking this option included farmers with young lambs on farm. These findings suggest that sheep scab will be dealt with whenever the disease appears on the farm and that dipping is an option that is palatable to the farming community all year round. The last dipping was done by the end of March 2021 and treatment was completed on all farms with 2 weeks of the diagnosis and again this would have helped with reducing onward spread.

Of the farms that had negative results on testing with the ELISA a number had previously treated their sheep. Interpretation of the ELISA results was considered prior to the sampling. Where the animals were in the active period of the product administered, then antibodies were considered indicative of an infestation that had been treated before clinical signs were apparent, as part of the farms routine management and required no further action by the programme. Similarly where antibodies were detected in the 2‐month period after the end of the active period of the product, then this was considered as evidence of infestation that had been treated. Where the time between treatment and blood sampling was over 4 months then a positive antibody test result was regarded as indicative of a new infestation.^7^ None of the farms with prior treatment had sheep with antibodies, so no further action was indicated on these farms. It is possible that the test misclassified an infested farm as clear, but this is thought to be unlikely.

A further question that was raised in discussion with the veterinary surgeons and farmers was the handling of animals that were near to market. It was a concern of the veterinary surgeons that lambs due to go to slaughter might be delayed, due to the withdrawal periods of the treatments administered, leading to increased costs to the farmer. Therefore, a balance between the use of antiparasiticides and the option of sending animals for immediate slaughter may be a more appropriate response to the presence of scab in this class of sheep.

This pilot project suggests that local cooperation can be an effective way of dealing with sheep scab in the local community. It does however require external input from veterinary surgeons and possibly other coordinating groups to achieve the results as described in this report. The further investigation of onward transmission and long‐term prevention of sheep scab require further consideration to ensure that the maximum benefits from this approach are realised. Even without this knowledge the authors commend this approach to veterinary surgeons in practice as a more sustainable, farmer‐led, means of controlling sheep scab in the future.

## CONFLICTS OF INTEREST

The authors declare they have no conflicts of interest.

## ETHICS STATEMENT

Informed consent was gained from all of the participants, before involvement in the programme, including staff from the general veterinary practices and the farmers.

## Data Availability

The data that support the findings of this study are available from the corresponding author upon reasonable request.

## References

[1] van den Broek AH , Huntley JF . Sheep Scab: the disease, pathogenesis and control. J Comp Path. 2003;128(2):79–91. 12634083 10.1053/jcpa.2002.0627

[2] Kirkwood AC . Effect of *Psoroptes ovis* on the weight of sheep. Vet Rec. 1980;107(20):469–70. 7445347 10.1136/vr.107.20.469

[3] Nixon EJ , Wall R , Vineer HR , Stubbings L . The high cost of sheep scab. Vet Rec. 2020;187(8):325 (Opinion). 10.1136/vr.m3888 33060237

[4] Cross P , Edwards‐Jones G , Omed H , Williams AP . Use of a randomized response technique to obtain sensitive information on animal disease prevalence. Prev Vet Med. 2010;96(3):252–62. 20580109 10.1016/j.prevetmed.2010.05.012

[5] Welsh Government . Available from: https://gov.wales/sites/default/files/publications/2022‐01/animal‐health‐and‐welfare‐framework‐implementation‐plan‐2022‐2024.pdf. Accessed 27 Jan 2022.

[6] Bates PJ Sheep scab (*Psoroptes ovis*). In: Aitken ID, editor. Diseases of Sheep. 4th ed. Oxford, UK: Blackwell Publishing; 2007. p. 321–5.

[7] Burgess ST , Innocent G , Nunn F , Frew D , Kenyon F , Nisbet AJ , et al. The use of a *Psoroptes ovis* serodiagnostic test for the analysis of a natural outbreak of sheep scab. Parasit Vectors. 2012;5:7. 22233730 10.1186/1756-3305-5-7 PMC3266638

[8] Hamer K , Burgess S , Busin V , Sargison ND . Performance of the *Psoroptes ovis* antibody enzyme‐linked immunosorbent assay in the face of low‐level mite infestation. Vet Rec. 2019;185(4):107. 10.1136/vr.105304 31127028

[9] O'Brien DJ . Treatment of psoroptic mange with reference to epidemiology and history. Vet Parasitol. 1999;83(3–4):177–85. 10.1016/s0304-4017(99)00056-4 10423001

[10] Beynon SA . Potential environmental consequences of administration of ectoparasiticides to sheep. Vet Parasitol. 2012;189(1):125–35. 22538092 10.1016/j.vetpar.2012.03.041

[11] Coggon D . Work with pesticides and organophosphate sheep dips. Occup Med. 2002;52(8):467–70. 10.1093/occmed/52.8.467 12488517

[12] Jacobs CT , Scholtz CH . A review on the effect of macrocyclic lactones on dung‐dwelling insects: toxicity of macrocyclic lactones to dung beetles. Onderstepoort J Vet Res. 2015;82(1):858. 10.4102/ojvr.v82i1.858 26017637 PMC6238710

[13] Doherty E , Burgess S , Mitchell S , Wall R . First evidence of resistance to macrocyclic lactones in *Psoroptes ovis* sheep scab mites in the UK. Vet Rec. 2018;182(4):106. 10.1136/vr.104657 29317477

[14] Sturgess‐Osborne C , Burgess S , Mitchell S , Wall R . Multiple resistance to macrocyclic lactones in the sheep scab mite *Psoroptes ovis* . Vet Parasitol. 2019;272:79–82. 31395209 10.1016/j.vetpar.2019.07.007

[15] Nunn FG , Burgess STG , Innocent G , Nisbet AJ , Bates P , Huntley JF . Development of a serodiagnostic test for sheep scab using recombinant protein Pso o 2. Mol Cell Probes. 2011;25(5):212–8. 21963943 10.1016/j.mcp.2011.09.002

[16] Welsh Government . £5m to help eradicate sheep scab, 2019. Available from: https://gov.wales/ps5m‐help‐eradicate‐sheep‐scab. Accessed 24 Jan 2022.

